# The Role of Exosomal Non-Coding RNAs in Coronary Artery Disease

**DOI:** 10.3389/fphar.2020.603104

**Published:** 2020-12-08

**Authors:** Jia Liu, Junduo Wu, Longbo Li, Tianyi Li, Junnan Wang

**Affiliations:** Department of Cardiology, The Second Hospital of Jilin University, Changchun, China

**Keywords:** exosomes, Non-coding RNAs, coronary artery disease, atherosclerosis, acute myocardial infarction

## Abstract

Cardiovascular disease (CVD) remains the leading cause of morbidity and mortality worldwide. Atherosclerosis (AS) is a major cause of CVD. Oxidative stress, endothelial dysfunction, and inflammation are key factors involved in the development and progression of AS. Exosomes are nano-sized vesicles secreted into the extracellular space by most types of cells, and are ideal substances for the transmission and integration of signals between cells. Cells can selectively encapsulate biologically active substances, such as lipids, proteins and RNA in exosomes and act through paracrine mechanisms. Non-coding RNAs (ncRNAs) are important for communication between cells. They can reach the recipient cells through exosomes, causing phenotypic changes and playing a molecular regulatory role in cell function. Elucidating their molecular mechanisms can help identify therapeutic targets or strategies for CVD. Coronary artery disease (CAD) is the most important disease in CVD. Here, we review the role and the regulatory mechanism of exosomal ncRNAs in the pathophysiology of CAD, as well as the potential contribution of exosomal ncRNA to diagnosis and treatment of CAD.

## Introduction

In the past 20 years, the prevalence of cardiovascular disease (CVD) has increased dramatically, becoming one of the major concerns worldwide. Atherosclerosis (AS) is a major pathological cause of CVDs, including ischemic heart disease, stroke, heart failure, hypertension and peripheral vascular diseases. It is the leading cause of death worldwide and imposes a huge social and economic burden (Plakht et al., 2015; Roth et al., 2017; Benjamin et al., 2017). Coronary artery disease (CAD) is the most important disease in CVD. Although the development of drugs and coronary interventional therapy has greatly improved the clinical prognosis (Neumann et al., 2019), patients with CAD still face several problems, such as stent restenosis, cardiac remodeling, and ischemia/reperfusion injury. Therefore, studying the pathogenesis of CAD and developing new and reliable treatment methods is of great significance. Oxidative stress, endothelial dysfunction, and inflammation are involved in the development and progression of AS (Gimbrone Jr and García-Cardeña, 2016; Kattoor et al., 2017; Marchio et al., 2019). Studies on the pathophysiology of AS would help improve the diagnosis and treatment of CAD.

Non-coding RNAs (ncRNAs), mainly including microRNAs (miRNAs), long ncRNAs (lncRNAs), and circular ncRNAs (circRNAs), have attracted increasing attention in recent years. They play an important role in the development of CVD through different mechanisms (Poller et al., 2018; Fasolo et al., 2019). Especially, the binding of ncRNA and messenger RNA (mRNA) and their regulatory mechanism has gained great attention. The main action of miRNAs is the negative regulation of gene expression by binding to a target mRNA and inducting its degradation or inhibiting of its translation (Beermann et al., 2016). MiRNAs function as signaling molecules transferring genetic information between cells or tissues (Viereck et al., 2014). For example, miRNAs derived from adipose in the circulation (similar to hormones) may serve as a genetic form of adipokines for regulating metabolism in distant organs (Fatima and Nawaz, 2017a). LncRNAs and circRNAs function as sponges, which affect the expression of related mRNAs through their interaction with miRNAs, establishing the mechanism of competitive endogeneous RNA. In addition, lncRNAs have diverse functions as signals, decoys, guides, and scaffolds (Wang and Chang, 2011; Fatima and Nawaz, 2017b). NcRNAs are involved in apoptosis, autophagy, necrosis, fibrosis, angiogenesis, migration, proliferation, and other pathophysiological processes in myocardiocytes and vascular endothelial cells, myocardial fibroblasts and Vascular smooth muscle cells (VSMCs) under stress. They also regulate neointima formation, lipid metabolism, atherosclerosis process in the occurrence and development of CVD. Circulating ncRNAs are increasingly recognized as potential biomarkers for risk stratification, diagnosis and prognosis of cardiac injury, therapeutic targets, and multiple forms of cardiovascular disease (Barwari et al., 2016; Viereck and Thum, 2017).

Exosomes are nano-sized membrane vesicles which carry biomolecules including DNA, mRNA, ncRNAs, lipids and proteins. Exosomes, which are major players in intercellular communication, released from different cell types transport different substances to target cells, enable information exchange between adjacent cells or distant cells, and play a biological function. For example, VSMC-derived exosomes mediate the transfer of miR-155 from VSMCs to endothelial cells (ECs), destroying tight junctions and the integrity of endothelial barriers, leading to an increased endothelial permeability and enhanced atherosclerotic progression (Zheng et al., 2017).

CAD is a common condition that strongly correlates with increased cardiovascular morbidity and mortality. Exosomal ncRNAs play crucial roles in intercellular communication under physiological and pathological conditions and are recognized as readily accessible biomarkers for diagnosis and prognosis of CAD. This review focuses on the role of exosomal ncRNAs in the pathogenesis of CAD and their key roles in the prevention and treatment of CAD.

### Exosomes

#### Exosome Biogenesis

**FIGURE 1:**
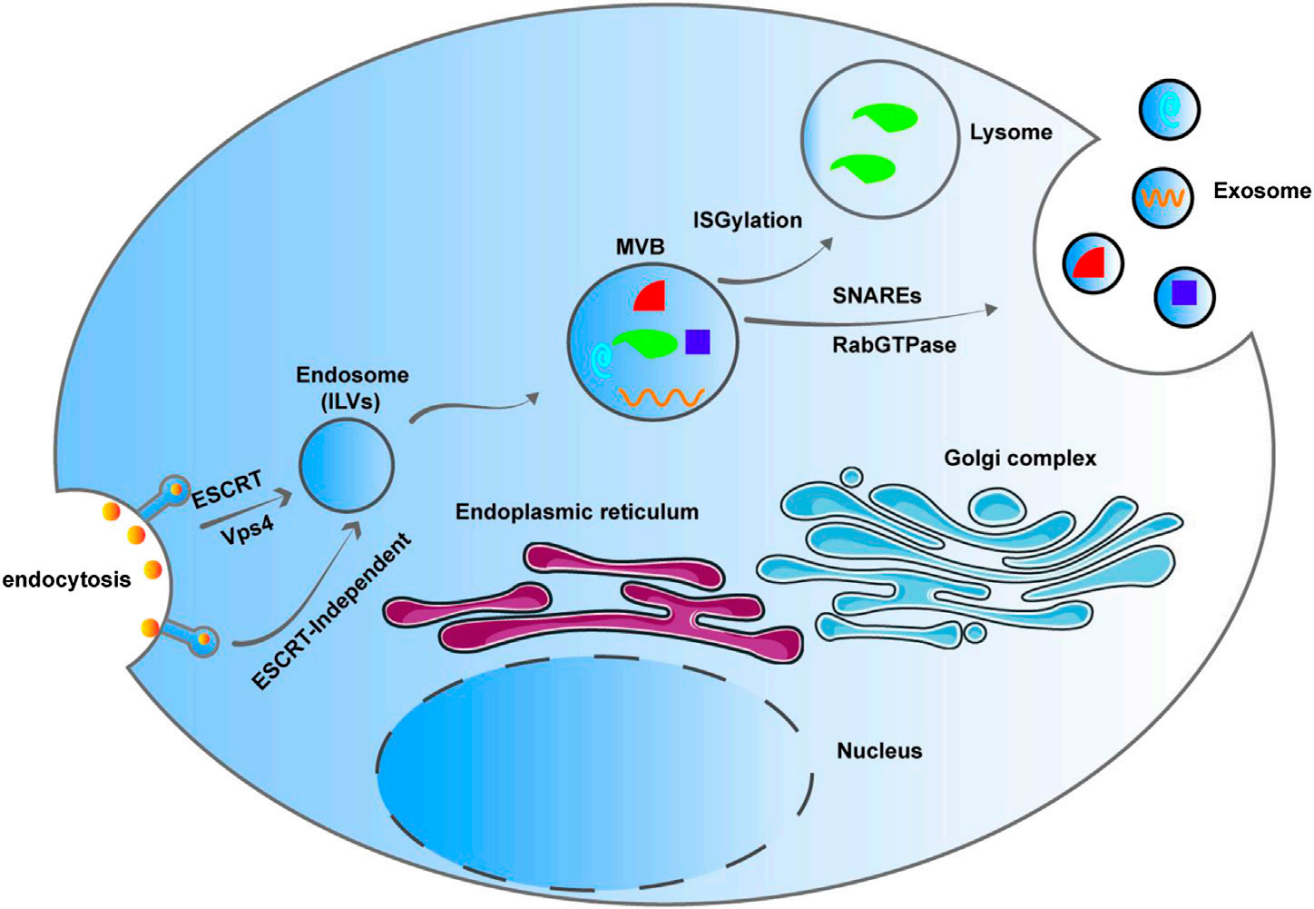
Intraluminal vesicle (ILV) are formed through invagination of the endosome. ILV containing endosome changes into multivesicular bodies (MVBs), which are also called late endosomes, through Endosomal sorting complexes required for transport (ESCRT) pathway and Vps4. MVBs participate degradation through ISGylation-mediated fusion with lysosomes. MVBs fuse with the plasma membrane through Rab-GTPase and SNARES to form exosomes and release them into the extracellular space, attaining communication between cells. Melanosomes and exosomes exhibit ESCRT-independent ILV formation that is dependent on selforganizing lipid and cargo domains.

Exosomes are bilayer membrane vesicles that were originally described by Pan and Johnstone (1983). They are phospholipid-coated structures, cup-shaped under electron microscope, varying in size from 30 to 150 nm (Xu et al., 2015). Exosomes are smaller than other extracellular vesicles like apoptotic bodies and microvesicles. They are membrane-bound organelles released by different types of cells and contain many bioactive molecules, such as proteins, lipids, and nucleic acids. Vesicles vary in size, biogenesis, and content, and usually include apoptotic bodies, microvesicles, and exosomes (Théry, 2011; Boulanger et al., 2017). Intraluminal vesicles (ILVs) are formed through invagination of the endosome. ILV containing endosome changes into multivesicular bodies (MVBs), which are also called late endosomes, through endosomal sorting complexes required for transport (ESCRT) and vacuolar protein sorting 4 (Vps4) pathway. On the one hand, MVBs play a role in protein degradation through ISGylation-mediated fusion with lysosomes. On the other hand, MVBs fuse with the plasma membrane through Rab-GTPase and SNARES to form exosomes and release them into the extracellular space, attaining communication between cells. Melanosomes and exosomes exhibit ESCRT-independent ILVs formation that is dependent on selforganizing lipid and cargo domains (Babst, 2011) (Figure 1). Most body fluids, such as plasma, saliva, serum, breast milk, amniotic fluid, and urine, contain significant amounts of exosomes (Simons and Raposo, 2009), and they are released into the culture medium by cultured cells *in vitro* (Sun et al., 2017).

#### Intracellular Communication

Intercellular communication plays an important role in maintaining the survival and dynamic balance of multicellular systems. Communication between cells is achieved by direct contact or secretion of signaling molecules (Nassiri and Rahbarghazi, 2014). Exogenous components, to a large extent, reflect the physiological or pathological state of the cell, which means that the secretion, content, and function of exosomes will change with the changing cell microenvironment. Several studies have identified an important paracrine cell-cell communication mechanism involved in the extracellular transfer and its subsequent effects on receptor cell function (Lawson et al., 2016). Exosomes are ideal substances for transmitting and integrating signals between cells, as they are released from different cell types, and contain different biologically active molecules, including lipid, protein, and nucleic acids. Once secreted, exosomes can enter neighboring target cells or travel into the body fluids to reach distant cells. Exosomes bear surface molecules that allow them to be specifically targeted to other cells. Once attached to a recipient cell, exosomes can induce signaling through receptor-ligand interaction or can be internalized via endocytosis, thereby altering the physiological state of the recipient cell and playing a biological function (Musante et al., 2014). For example, macrophages in arteriosclerotic lesions can interact with VSMCs via exosomes, thereby inducing VSMCs migration and adhesion to the intima. Another study has shown that MSC-derived exosomes modified by TIMP2 can repair ischemic myocardium by inhibiting myocardial cell apoptosis, promoting angiogenesis, and remodeling extracellular matrix (Ni et al., 2019). However, exosomal ncRNAs also influence CVD phenotype. For example, VSMC-derived exosomes mediate the transfer of miR-155 from VSMCs to ECs, destroying tight junctions and the integrity of endothelial barriers and leading to an increased endothelial permeability and enhanced atherosclerotic progression (Zheng et al., 2017). Different stages of tissue damage can be identified by measuring bioactive molecules in circulation. Exosomes can also enrich signaling molecules to display them at a high partial concentration. They also contain cytokines, pathogen-associated molecular patterns and damage-associated molecular patterns, and autoantigens. The double-layer membrane seal environment of exosomes protects signaling molecules from being degraded by enzymes. Therefore, exosomes have attracted attention in the field of cardiovascular medicine as regulators of pathophysiological processes, diagnostic markers, and therapeutic candidates.

#### Exosome Isolation

Several methods have been used to isolate and analyze exosomes, including ultracentrifugation, ultrafiltration, immune affinity capture, and microfluidics-based isolation techniques (Witwer et al., 2013; Szatanek et al., 2015; Boriachek et al., 2018). Ultracentrifugation includes analytical and preparative ultracentrifugation (Li et al., 2017). Analytical ultracentrifugation is used to study the physical and chemical properties of granular materials and the intermolecular interactions of polymer materials. There are two types of preparative ultracentrifugation: differential ultracentrifugation and density gradient ultracentrifugation. Preparative hypervelocity centrifugation plays an important role in exosome separation. It can be used to separate small biological particles, such as bacteria, viruses, organelles, and extracellular vesicles. Ultracentrifugation, considered as the gold standard, is the most commonly used method for exosome separation (Livshits et al., 2016), accounting for 56% of all exosome separation technologies adopted. However, this method has some limitations, such as time consumption, large sample size, and low recovery rate, which is not suitable in clinical practice (Zarovni et al., 2015). Size-based isolation techniques include ultrafiltration, size exclusion chromatography, flow-field-flow fractionation (F4), and hydrostatic filtration dialysis (HFD) (Musante et al., 2014). Ultrafiltration is based on the size of exosomes using a membrane filter with a defined molecular weight or size exclusion limit to separate exosomes. It is faster than ultracentrifugation, and it does not require special equipment. However, excessive force can cause large vesicles to deform and break, affecting the results of the experiment. A commercial exosome isolation kit has been developed for exosome isolation and RNA extraction from isolated exosomes using the rapid filter separation process (Macías et al., 2019), which can be extracted from cell-free samples, such as the serum, urine, cerebrospinal fluid, and cell culture medium. Techniques based on immune affinity capture include a microplate ELISA (Zarovni et al., 2015) and magnetic immunocapture of submicron magnetic particles.

Over the past 20 years, the molecular composition of exosomes from different sources has been studied extensively (Raimondo et al., 2011; Choi et al., 2015). Protein, RNA, and lipid content of molecules in exosomes vary greatly depending on the type of parent cells and the microenvironment (Zaborowski et al., 2015; Skotland et al., 2017). In general, exosomes are composed of the following molecules: Tetraspanin family (CD9, CD63, and CD81 transmembrane proteins); tumor susceptibility gene 101 (TSG101); major histocompatibility complex (MHC) II class of molecules; programmed cell death 6-interacting protein (PDCD6IPs) (Lötvall et al., 2014; Théry et al., 2018). In addition, heat shock proteins (HSP60, HSP70, and HSP90), cytoskeleton proteins, fibrinolytic protein-1, membrane coupling proteins, and membrane transporters are present in exosomes, regardless of the type, and these proteins are commonly used as exosome markers. Currently, the ongoing studies on exosomes employ a combination of transmission electron microscopy, western blotting (or flow cytometry), and nanoparticle tracking analyses, which are the most widely used methods to detect exosomes.

Increasing evidence shows that exosomes released by ECs, smooth muscle cells, fat cells, platelets, cardiomyocytes, and stem cells play an important role in the occurrence and development of CVD (Barile et al., 2018; Sluijter et al., 2018; Gao et al., 2019). Exosomes from different cell types are involved in angiogenesis and cell migration, proliferation, apoptosis, hypertrophy, and regeneration. In the hypoxic state, the contents and quantity of exosomes in the myocardiocytes change significantly (Emanueli et al., 2016; Li H. et al., 2019; Li J. et al., 2019; Li N. et al., 2019), suggesting that the injured myocardium can release specific exosomes into body fluids. Therefore, the detection of exosomal cargos reflects the pathophysiology of cardiomyocytes.

### Non-Coding RNAs

Less than 2% of the human genome encodes known proteins (ENCODE Project Consortium, 2012). The ENCODE Project found that intergene regions could be transcribed and more than 85% of the genome was transcribed into ncRNAs. Whether these ncRNAs are functional needs further study (Hangauer et al., 2013). Initially, ncRNAs were considered as transcriptional noise or residual waste generated during RNA processing. Recently numerous studies have shown that ncRNAs are indispensable functional molecules in most cells, which regulate the pathophysiology of CVD through various mechanisms, and ncRNAs can be used for the diagnosis and prevention of CVDs (Poller et al., 2018). NcRNAs are involved in apoptosis, autophagy, necrosis, fibrosis, proliferation, and migration of cardiomyocytes, ECs, cardiac fibroblasts, and VSMCs, leading to CVD (Fasolo et al., 2019).

NcRNAs mainly include lncRNAs, circRNAs and miRNAs. MiRNAs are endogenous ncRNAs that are approximately 18–24 nucleotides in length and play an important role in the regulation of protein biosynthesis. Several physiological processes and pathological outcomes are highly dependent on miRNAs, including cancer, CVDs, and neurological diseases (Lekka and Hall, 2018). Recently, accumulating evidence has shown that miRNAs may serve as important new biomarkers that can be used for CVD diagnosis in the future (Ding et al., 2019; Zhang L. et al., 2020; Zhang L. et al., 2020). The effect of miRNAs on mRNA expression is often cellular or organ-specific, highly dependent on the stress and metabolic state of the organism, and usually not correlated with miRNA expression levels. It shows that various regulatory mechanisms not only control their expression but also control their activity and bioavailability (Anfossi et al., 2018). Compared with small ncRNAs, lncRNAs are more heterogeneous and are difficult to classify and characterize. LncRNAs are localized in the nucleus and cytoplasm. Unlike miRNAs or proteins, specific functions of lncRNAs are not easily inferred from their sequence or structure. LncRNAs and circRNAs are over 200 nucleotides in length (Bär et al., 2016). LncRNAs are associated with many diseases, including CVD (Uchida and Dimmeler, 2015; Gomes et al., 2018). CircRNAs are evolutionarily conserved, stable, endogenous, type-specific, and tissue-specific molecules that are produced via reverse splicing events. CircRNAs have a variety of biological functions, including miRNA sponging, protein sponging, regulation of transcription, splicing interference, and possess a minimal protein-coding ability (Uchida and Dimmeler, 2015).

## Exosomal Non-Coding RNAs in the Pathophysiology of Coronary Artery Disease

**FIGURE 2:**
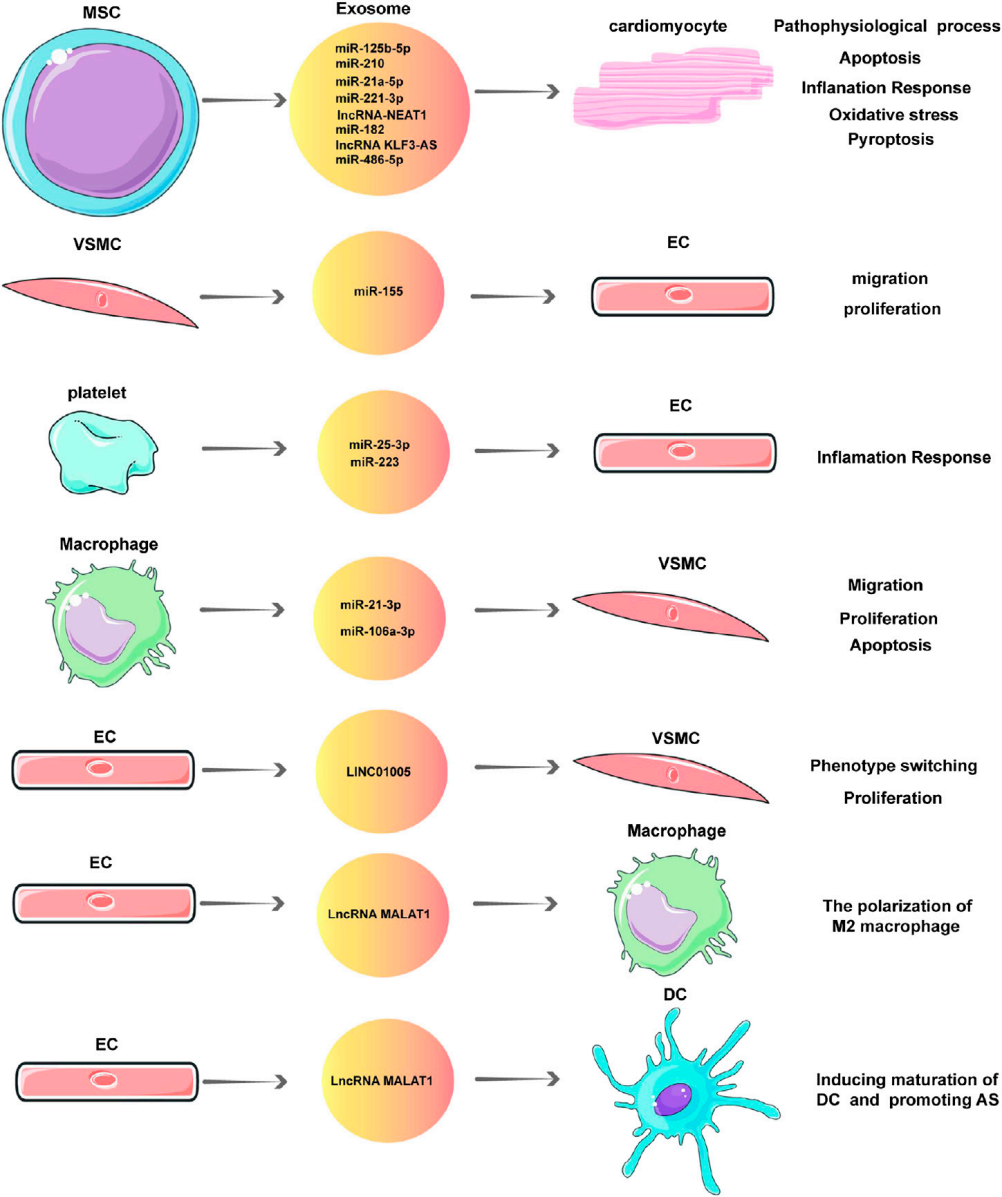
Exosomal non-coding RNAs (ncRNAs) in the pathophysiology of coronary artery disease (CAD).

Pathophysiological processes such as apoptosis, autophagy, proliferation, migration, and fibrosis occur when myocardial cells, ECs, myocardial fibroblasts, and VSMCs are under stress, which is involved in the occurrence and development of CVDs. NcRNAs in exosomes participate in these pathophysiological processes and play an important role in CAD (Table 1; Figure 2). This section focuses on the pathophysiological role of exosomal ncRNAs in CAD.

**TABLE 1:** Exosomal ncRNAs in coronary artery disease.

Exosomal ncRNA	Exosome source	Target cell	Pathway	Protective effects	References
miR-155↑	VSMC	EC	KLF5	Promote migration and proliferation of EC	Zheng et al. (2017)
LINC01005↑	HUVEC	VSMC	KLF4/miR-128-3p	Promote phenotype transformation, proliferation and migration of VSMC	Zhang Z. et al. (2020)
miR-21-3p↑	THP-1 macrophage	VSMC	PTEN	Promote migration and proliferation of VSMC	Zhu et al. (2019)
miR-106a-3p↑	THP-1 macrophage	VSMC	CASP9/caspase	Promote proliferation and inhibit apoptosis of VSMC	Liu et al. (2020)
miR-221-3p↑	BMSC	Myocardial cells	PTEN/Akt	Promote angiogenesis, migration and proliferation, inhibit apoptosis of myocardial cells	Sun et al. (2020)
miR-486-5p↑	BMSC	H9C2	PTEN/PI3K/AKT	Inhibit apoptosis of H9C2	Sun et al. (2019)
miR-214↑	BMSC	CSC	CaMKII	Inhibit oxidative stress, apoptosis, and Ca2+ homeostasis	Wang Y. et al. (2018)
miR-21a-5p↑	MSC	Myocardial cells	PDCD4,PTEN, Peli1,FasL	Inhibit apoptosis of myocardial cells	Luther et al. (2018)
lncRNA AK139128↑	Cardiomyocytes	Fibroblasts	N/A	Promote cell apoptosis, inhibit cell proliferation	Wang and Zhang (2020)
miR-24↑	BMSC	Myocardial cells	bax,caspase-3	Inhibit apoptosis	Zhang C. S. et al. (2019)
miR-125b-5p↑	BMSC	Myocardial cells	p53,BAK1	Inhibit apoptosis	Zhu L. P. et al. (2018)
miR-210↑	MSC	Cardiomyocytes	—	Inhibit apoptosis of cardiomyocytes, reduce fibrosis	Zhu J. et al. (2018)
lncRNA GAS5↑	THP-1	EC	N/A	Promote apoptosis of vascular endothelial cells	Chen et al. (2017)
miR-221/222↑	HAOSMC	HUVEC	PTEN/Akt	Inhibit autophagy of HUVECs	Li et al. (2016)
miR-30a↑	Myocardial cells	Myocardial cells	BECLIN-1,ATG12,LC3II/LC3I	Promote autophagy	Yang et al. (2016)
miR-125b-5p↑	BMSC	NMCM	p53-Bnip3	Improve autophagy flux	Xiao et al. (2018)
miR-126↑	Adipose stem cells	H9C2	—	Reduce damage of H9c2 myocardial cell	Luo et al. (2017)
miR-let7↑	MSC	M2 macrophage	HMGA2/NF-κB,IGF2BP1/PTEN	Promote M2 macrophage polarization and inhibit macrophage infiltration	Li J. et al. (2019)
miR-125b↑	BMSC	Myocardial cells	SIRT7	Inhibit apoptosis of myocardial cells and inflammatory response	Chen Q. et al. (2020)
miR-146a↑	ADSC	Myocardial cells	TLR4/NFκB,EGR1	Inhibit myocardial apoptosis, inflammatory response, and fibrosis	Pan et al. (2019)
lncRNA KLF3-AS1↑	BMSC	Myocardial cells	Sirt1/miR-138-5p	Inhibit pyroptosis	Mao et al. (2019)
miR-25-3p↑	Platelet	EC	ADAM10	Inhibit inflammation and lipid deposition of EC	Yao et al. (2019)
miR-223↑	Mononuclear cells	HUVECs	N/A	Inhibit inflammation of EC	Liu et al. (2018)
lncRNA MALAT1↑	EC	M2 macrophages	N/A	Promote the polarization of M2 macrophages and anti-atherosclerosis	Huang et al. (2018)
miR-182↑	MSC	Myocardial cells	N/A	Reduce the myocardial I/R damage	Geng et al. (2020)
miR-423-3p↑	Myocardial fibroblasts	H9C2	RAP2C	Anti-inflammatory	Fu et al. (2018)
lncRNA MALAT1↑	HUVEC	DC	Nrf2	Induce maturation of DC cells and promote AS	Li H. et al. (2019)
miR - 146a↑	Dendritic cells	HUVEC	IRAK	Protect HUVECs from stimulation	Zhong et al. (2019)
miR-223↑	Platelet	EC	ICAM-1,NF-κB,MAPK	Anti-inflammation of EC	Li J. et al. (2019)
miR-204/miR-211↑	VSMC	VSMC	N/A	Alleviate vascular calcification and senescence	Xu et al. (2020)
miR-21↑	MSC	CSC	PTEN,PI3K/AKT	Protect CSC from cell death	Shi et al. (2018)
miR-210↑	MSC	Cardiomyocytes	AIFM3	Protect cardiomyocytes from stress	Cheng et al. (2020)
lncRNA-NEAT1↑	MSC	Cardiomyocytes	miR-142-3p/FOXO1	Protect cardiomyocytes from apoptosis	Chen H. et al. (2020)
circHIPK3↑	Cardiomyocytes	EC	miR-29a/IGF-1	Inhibit oxidative stress-induced CMVEC dysfunction and promote proliferation, migration, and tube formation of EC	Wang et al. (2020), Wang Y. et al. (2019)

ncRNAs, non-coding RNAs; (↑) and (↓) indicate increased and decreased expression of ncRNA respectively.

### Migration and Proliferation

Cell proliferation and migration are necessary for the formation of atherosclerotic plaques in the development of AS. ECs are exposed to various destructive stimuli, such as oxidized low-density lipoprotein (ox-LDL), which leads to vascular endothelial impairment. VSMC activation and macrophage infiltration contribute to the formation of atherosclerotic plaque. KRüppel-like factor5 (KLF5) is a transcription factor which plays a core role in cardiovascular remodeling by mediating the proliferation and migration of VSMCs. It was found that exosomes derived from VSMCs mediate KLF5-induced miR-155 transfer from VSMCs to ECs; inhibit EC proliferation, migration, and re-endothelialization; and further damage tight connections and endothelial barrier integrity, leading to increased endothelial permeability and accelerated AS process (Zheng et al., 2017). A previous study reported that VSMC phenotype transformation plays an important role in the development of AS (Bennett et al., 2016). *In vivo* studies by Zhu et al. showed that nicotine-induced atherosclerotic lesion progression leads to exosome retention in plasma. At the same time, VSMCs co-cultured with nicotine-stimulated macrophages shows strong migration and proliferation abilities, which were dependent on exosomes. MiR-21-3p is highly expressed in exosomes released from nicotine-treated macrophages, and it promotes VSMC migration and proliferation by directly targeting PTEN, thus accelerating the development of AS (Zhu et al., 2019). Liu et al. found that miR-106a-3p expression in the exosomes derived from ox-LDL induces the increase in THP-1 macrophages; these exosomes directly bind to CASP9 and inhibit the caspase signaling pathway, thus promoting the proliferation of VSMCs (Liu et al., 2020). Another study revealed that miRNAs derived from extracellular vesicles of atherogenic macrophages, especially miR-146a, may accelerate the development of AS by reducing cell migration and promoting macrophage embedding on the vascular wall (Nguyen et al., 2018).

Another study showed that LINC01005 in exosomes, derived from human umbilical vein endothelial cells (HUVECs) treated with ox-LDL, promote KLF4 expression through competitive binding to miR-128-3p, thus promoting phenotypic transformation, proliferation, and migration of VSMCs, and the development of AS (Zhang L. et al., 2020; Zhang L. et al., 2020).

In a study on the cardial protection of bone marrow mesenchymal stem cells (BMSCs), Sun et al. found that miR-221-3p in exosomes secreted from aged mesenchymal stem cells attenuates the angiogenic function of myocardial cells and promotes survival. Upregulation of miR-221-3p improves angiogenesis, migration, and proliferation of myocardial cells (Sun et al., 2020).

### Apoptosis

Apoptosis is a form of programmed cell death process controlled by genes to maintain homeostasis, and it plays a pivotal role in the pathogenesis of CVDs. Liu et al. found that ox-LDL induces an increase in miR-106a-3p in the exosomes of THP-1 macrophages, which can directly bind to CASP9 and inhibit the caspase signaling pathway and apoptosis of VSMCs (Liu et al., 2020). Chen et al. found lncRNA GAS5 in exosomes derived from THP-1 cells after ox-LDL stimulation. The exosomes derived from the overexpression of lncRNA-GAS5 in THP-1 cells are transported to vascular ECs and promote the apoptosis of vascular ECs, while lncRNA GAS5 knockout in the exosomes inhibits the apoptosis of ECs. This suggests that lncRNA GAS5 regulates the apoptosis of macrophages and ECs through exosomes in AS development (Chen et al., 2017).

In an ischemia-reperfusion (I/R) model, BMSC-derived exosomes induce H9C2 cell proliferation and reduce apoptosis. Further experiments showed that miR-486-5p in exosomes derived from mesenchymal stem cells (MSC-exo) inhibits hypoxia/reoxygenation (H/R)-induced apoptosis of H9C2 cells. Experiments confirmed that MSC-exo inhibits the expression of PTEN in H9C2 cells through miR-486-5p, and then activates the PI3K/AKT pathway to inhibit the apoptosis of damaged cardiomyocytes and repair the myocardial injury caused by I/R (Sun et al., 2019). Exosomes of BMSCs promote cell proliferation and survival through the transport of various bioactive molecules, including miRNA. Wang et al. found that when exosomes derived from BMSCs are pretreated with cardiac stem cells (CSCs), exosomes are efficiently absorbed, and exosomal miR-214 is transferred into CSCs to participate in cell signal transduction pathways. This transfer successfully induces downstream responses in CSCs, and decreases CaMKII expression, oxidative stress, apoptosis, and Ca^2+^ homeostasis. This study indicated that the extracellular miR-214 derived from BMSCs plays a key role in the regulation of apoptosis (Wang Y.et al., 2018; Wang Z. et al., 2018). Sun et al. found that miR-221-3p in exosomes secreted from aged BMSCs attenuates the angiogenic function of myocardial cells and promotes the survival of myocardial cells. Upregulation of miR-221-3p inhibits cell apoptosis through PTEN/Akt pathway (Sun et al., 2020). Similarly, Luther et al. proved that exosomes in mesenchymal stem cells (MSCs) downregulate the expression of pro-apoptotic gene products PDCD4, PTEN, Peli1, and FasL in cardiomyocytes by increasing the expression of miR-21a-5p in the recipient cardiomyocytes, thus inhibiting apoptosis (Luther et al., 2018). Another study confirmed that exosomes obtained from pretreated hypoxic BMSCs inhibit apoptosis of myocardial cells in rats with acute myocardial infarction (AMI) by upregulating miR-24 (Zhang C. S. et al., 2019; Zhang Y. G. et al., 2019). Zhu et al. confirmed that miR-125b-5p in exosomes derived from BMSCs under hypoxic conditions promote ischemic heart repair by inhibiting apoptosis in myocardial cells (Zhu et al., 2018). Another study revealed that miR-21 in exosomes derived from cardiac progenitor cells protects cardiomyocytes from oxidative stress-related apoptosis by downregulating PDCD4 (Xiao et al., 2016). Hypoxia is an important trigger of myocardial remodeling during the development of CVD. In a study on the effect of exosomes on cardiac fibroblasts, it was found that hypoxia upregulates the expression of lncRNA AK139128 in cardiomyocytes and exosomes, and lncRNA AK139128 in exosomes derived from hypoxic cardiomyocytes promotes apoptosis, inhibits cell proliferation, and regulates fibroblast activity (Wang and Zhang, 2020).

### Autophagy

A recent study has shown that cardiovascular dysfunction and AS are closely related to autophagy (Grootaert et al., 2018). Autophagy is an intracellular process of self-digestion that typically removes depleted organelles and damaged cytoplasmic components from target cells through a bilipid bilayer membrane vesicle. Autophagy is considered to be a double-edged sword in the atherosclerotic inflammatory response (Hassanpour et al., 2019). Autophagy plays an important role in the pathogenesis of inflammation by affecting the homeostasis of inflammatory cells, including macrophages, neutrophils, and lymphocytes (Ding et al., 2013). Recent studies have shown that autophagy plays a vital role in the behavior of vascular cells, such as ECs and VSMCs. Li et al. found that exosomal miR-221/222 derived from human aortic smooth muscle cells (HAOSMCs) inhibits autophagy in HUVECs by partly regulating the PTEN/Akt signaling pathway when HUVECs and HAOSMCs are co-cultured (Li et al., 2016).

Several studies have shown that physiological autophagy plays a protective role in ischemic heart diseases (Kanamori et al., 2011). Autophagy is rapidly upregulated after AMI and promotes functional recovery after ischemia. MiR-30a was found to be highly enriched in the serum of patients with AMI and the cardiomyocyte culture upon hypoxia stimulation. It was also found that hypoxia-inducible factor-1 has a regulatory effect on miR-30a, which can be effectively transported between myocardial cells through exosomes upon hypoxia. Inhibition of miR-30a or the release of exosomes increases the expression of core autophagy regulators Beclin-1, ATG12 and LC3II/LC3I, thereby maintaining autophagy after hypoxia in myocardial cells. This study suggested that exosomes from hypoxic cardiomyocytes regulate autophagy via the paracrine transfer of miR-30a (Yang et al., 2016). Studies have demonstrated that transplantation of MSCs improves cardiac function and decreases infarct size after myocardial infarction in mice, and reduces apoptosis and autophagy flow. Under hypoxia and serum deprivation (H/SD) conditions, newborn mouse cardiomyocytes (NMCMs), when co-cultivated with MSCs or exosomes of MSCs, cause cell autophagy flux and a decrease in cell death and p53-Bnip3 signaling. Inhibiting the expression of miR-125b in exosomes could increase autophagy flux and cell death by regulating p53-Bnip3 signaling. The beneficial effect of BMSC transplantation after MI is partly due to the improved autophagy flux caused by the secretion of exosomes mainly containing miR-125b-5p (Xiao et al., 2018). *In vitro* experiments, cardiomyocytes can take up exosomes derived from myocardial microvascular ECs and increase Mst1 content, thereby inhibiting autophagy and promoting apoptosis of cardiomyocytes cultured in high glucose (Hu et al., 2018).

### Angiogenesis

At the site of myocardial ischemia during myocardial infarction, blood flow to the heart and oxygen are impaired. Angiogenesis occurs when arteries become narrow or are temporarily blocked, preventing oxygen-rich blood from reaching the heart. Angiogenesis of the heart after myocardial infarction is crucial for promoting the reperfusion and function of the ischemic heart. Sun et al. found that upregulation of miR-221-3p in exosomes secreted from MSCs improves angiogenesis of myocardial cells and promotes the survival of myocardial cells (Sun et al., 2020). Zhang et al. showed that exosomes derived from human umbilical cord mesenchymal stem cells (hucMSCs) accelerate angiogenesis via Wnt4/β/catenin signaling pathway (Zhang et al., 2015). Luo et al. found that exosomes derived from adipose stem cells overexpressing miR-126 significantly reduce the expression of inflammatory factors and H9c2 myocardial cell damage upon hypoxia. *In vivo* experiments showed that exosomes overexpressing miR-126 promote microangiogenesis and migration in rat myocardial infarction areas (Luo et al., 2017). Extracellular vesicles secreted by human-induced pluripotent stem cell-derived cardiomyocytes enhance angiogenesis in ECs, along with the increased expression of FGF2, VEGF2A, and PDGFA. This cell-free approach constitutes a potential approach for inducing angiogenesis in patients with myocardial infarction (Dougherty et al., 2018). Geng et al. found that miR-143 is significantly reduced in exosomes derived from the serum of patients with myocardial infarction, which promotes angiogenesis of myocardial ECs by targeting insulin-like growth factor 1 receptor (IGF-IR) and the production of NO (Geng et al., 2020).

### Fibrosis

Insufficient blood supply causes irreversible necrosis in cardiomyocytes after myocardial infarction. Since adult cardiomyocytes cannot proliferate, the necrotic areas of the myocardium are replaced by fibrous scarring (Frangogiannis, 2019). Fibroblasts play a key role in the formation of fibrous scars and are involved in different stages of heart tissue repair and play different roles (Fu et al., 2018). During the inflammatory phase, fibroblasts become active and acquire an inflammatory phenotype, thereby releasing inflammatory mediators to promote an inflammatory response. The matrix degradation activity of fibroblasts is activated to degrade extracellular matrix components. When cardiac repair enters the proliferative phase, the inflammatory response is inhibited, and most fibroblasts differentiate into myofibroblasts, which are characterized by anti-inflammatory effects, extracellular matrix production, and contractility. Myofibroblasts secrete extracellular matrix proteins to maintain the structural and functional integrity of the heart. Myofibroblasts also secrete anti-inflammatory factors to reduce inflammatory responses, which are beneficial to heart repair after myocardial infarction (Ma et al., 2017; Nakaya et al., 2017). To investigate whether exosomes derived from human umbilical cord mesenchymal stem cells (hucMSC-exo) promote the differentiation of myocardial fibroblasts into myofibroblasts in an inflammatory environment and protect myocardial cells. Shi et al. performed experiments which confirmed that hucMSC-exo increase the density of myofibroblasts in the infarcted area after myocardial infarction in the inflammatory stage, promote the differentiation of fibroblasts into myofibroblasts in the inflammatory environment, and reduce the inflammatory response. Fibroblast cultures pretreated with lipopolysaccharide (LPS) and exosomes from hucMSCs reduce apoptosis in myocardial cells (Shi et al., 2019). Some experiments have shown that specific miRNA overexpression in exosomes can inhibit myocardial fibrosis. Zhu et al. found that hypoxic culture augments miR-210 and nSMase2 activity in MSCs and their secreted exosomes. When exosomes derived from cultured MSCs under hypoxia were intramyocardially injected into the infarcted heart of mice, it resulted in significantly increased vascular density, lower myocardiocyte apoptosis, reduced fibrosis in the infarcted heart relative to those in the control group (Zhu et al., 2018). Another study showed that the level of miR-425 and miR-744 significantly decreased in the plasma exosome samples from patients with heart failure. The functional study confirmed that overexpression of miR-425 or miR-744 in cultured cardiac fibroblasts inhibits TGFβ1 expression and significantly inhibits angiotensin-induced collagen formation and fibrogenesis (Wang Y. et al., 2018; Wang Y. et al., 2018).

### Inflammatory Response

AS is recognized as a chronic lipid-induced inflammation of the vascular wall. Exosomes function as an important mediator of inter-cellular communication in the release of inflammatory cytokines and over-activation of inflammatory responses in AS (Hulsmans and Holvoet, 2013). Exosomes can be secreted by various type of cells in AS plaques and plasma. It has been found that platelet-derived exosomes could mediate platelet atherogenic interactions with ECs and monocytes (Goetzl et al., 2016). Another study indicated that exosomal miR-146a derived from oxLDL-treated macrophages promotes extracellular traps formation to drive AS (Zhang C. S. et al., 2019; Zhang Y. G. et al., 2019). However, there is no accurate conclusion about how cells secrete exosomes under specific conditions, and the exosome expression level in lesions and plasma is yet unavailable. The potential value of exosomes remains to be explored.

Exosomes from MSCs have immunomodulatory and immunosuppressive effects. It was found that treatment of ApoE^−/−^ mice with exosomes secreted by MSCs inhibits AS, promotes M2 macrophage polarization in plaques through the miR-let7/HMGA2/NF-κB pathway, and inhibits macrophage infiltration through the miR-let7IGF2BP1/PTEN pathway. This finding demonstrated that MSC-derived exosomes influence the inflammatory response to atherosclerotic plaques and provide a potential way to prevent AS (Li J. et al., 2019; Li N. et al., 2019).

Endothelial dysfunction is closely related to vascular EC injury and is involved in a series of pathophysiological processes. It is an important process triggering AS. Oxidized low-density lipoprotein (ox-LDL) induces ECs and macrophages to participate in the development of AS (Pirillo et al., 2013). The expression of miR-25-3p decreases in the vascular ECs and vascular tissues induced by ox-LDL. However, in the ApoE^−/−^ AS mouse model, miR-25-3p is highly expressed in platelet-derived exosomes, which downregulates ADAM10 expression and inhibits ox-LDL-induced vascular endothelial cell inflammation and lipid deposition (Yao et al., 2019). Chen et al. found that exosomes of vascular endothelial cell treated with ox-LDL aggravate AS by inducing the formation of neutrophil extracellular traps (Nets) through miR-505 in exosomes (Chen et al., 2019). Activated neutrophils release Nets with cytotoxic and thrombogenic effects, which play an important role in the pathological process of AS (Pertiwi et al., 2018). Liu et al. found that paeonol can inhibit EC signal transduction pathways, increasing the expression of exosomal miR-223 derived from mononuclear cells, thus decreasing the expression of IL-1, IL-6, VCAM-1, and ICAM-1 secreted from HUVECs, and decreasing the adhesion between mononuclear cells and ECs. This indicates the protective role of exosomal miR-223 derived from monocytes in the vascular endothelial cell inflammatory response (Liu et al., 2018). Xing et al. found that exosomes derived from fat stem cells inhibit the expression of miR-342-5p in the injury model, reversing the apoptotic effect of miR-342-5p on H_2_O_2_-induced HUVECs (Xing et al., 2020).

Research on hypercholesterolemic mice has shown that M2 macrophages reduce AS by preventing foam cell formation, and inhibiting M2 polarization promotes plaque progression (Fadini et al., 2014; Xu et al., 2017). Therefore, macrophage polarization plays an important role in the development of atherosclerotic plaques. Intramedullary injection of BMSC-derived exosomes after myocardial I/R injury in mice reduces the scope of myocardial infarction and the inflammation of the myocardium and serum. Exosomes derived from MSCs change the polarization state of macrophages by reducing miR-182 expression and myocardial I/R damage in mice, also inhibiting the inflammatory response (Zhao et al., 2019). Xie et al. found that exosomes derived from visceral adipose tissue of high-fat diet mice (HFD-VAT) significantly induce RAW264.7 macrophage M1 phenotype transformation and proinflammatory cytokine (TNF-α and IL-6) secretion, accompanied by the enhanced phosphorylation of NF-kB-p65. These results confirmed that exosomes derived from HFD-VAT promote AS by adjusting the polarization and the formation of macrophage foam cells, indicating that there is a new link between adipose tissue and AS in obesity (Xie et al., 2018). The maturation of dendritic cells regulates inflammation and contributes to the development of AS. Zhong et al. found that exosomes obtained from mature dendritic cells deliver miR-146a to HUVECs, and miR-146a helps protect HUVECs from the second stimulation by inhibiting IL-1 receptor-related kinases. These data suggest that there is a negative feedback loop in the inflammatory regulation of exosomes acquired by mature dendritic cells (Zhong et al., 2019). Li et al. found that the expression of miR-223, miR-339, and miR-21 in thrombin-activated platelet-derived exosomes increase. Thrombin-activated platelet-derived exosomes inhibit ICAM-1 expression during inflammation, and miR-223 may mediate this process by regulating NF-κB and MAPK pathways, demonstrating that platelet-derived exosomes are closely related to AS (Li et al., 2017). Another study found that the expression of miRNA-27a, miRNA-28-3p, and miRNA-34a was increased in the local myocardium of the infarction area upon myocardial infarction, which is largely expressed in exosomes released by cardiomyocytes and fibroblasts and involved in the imbalance of the Nrf2/ARE signaling pathway. This may promote oxidative stress in chronic heart failure by inhibiting the expression of Nrf2, thereby promoting the inflammatory response (Tian et al., 2018).

Huang et al. found that lncRNA MALAT1 in exosomes from ECs treated with ox-LDL promotes the polarization of M2 macrophages, playing a certain anti-AS role (Huang et al., 2018). Another study confirmed that the reduction of MALAT1 in exosomes, obtained from HUVECs and treated with ox-LDL, induces the maturation of DCs and promotes AS development (Li J. et al., 2019; Li N. et al., 2019). Wang et al. found that the expression of circHIPK3 is upregulated in the exosomes secreted by hypoxia-induced cardiomyocytes and that circHIPK3 sponges miR-29a. This upregulates the expression of IGF-1 and reduces the dysfunction of cardiac microvascular endothelial cells (CMVEC), induced by oxidative stress, and inhibits the inflammatory response (Wang Y. et al., 2019; Wang Z. et al., 2019).

Another study revealed that high expression of miR-125b in exosomes derived from BMSCs improves the survival rate of myocardial cells in rats after I/R by regulating SIRT7, inhibiting apoptosis of myocardial cells and inflammatory response, and improving cardiac function (Chen et al., 2020). Studies of exosomes derived from fat stem cells (ADSCs) have shown that miR-146a inhibits post-transcriptional EGR1 expression and reverses the activation of AMI, or hypoxia-induced TLR4/NF-κB signaling, inhibiting myocardial apoptosis, inflammatory response, and fibrosis. These results suggest that exosomes from miR-146a-modified ADSCs reduce AMI-induced myocardial injury by downregulating EGR1 (Pan et al., 2019). Mao et al. found that lncRNA KLF3-AS1 in exosomes secreted by BMSCs, as ceRNA, sponges miR-138-5p and regulates the expression of Sirt1, inhibiting pyroptosis, protecting hypoxic cardiomyocytes, and delaying the progression of myocardial infarction (Mao et al., 2019). During the acute phase of I/R injury and ischemic post-treatment, myocardial fibroblasts participate in cardiac protection through the exosome pathway and play an anti-inflammatory role by upregulating miR-423-3p expression in myocardial fibroblast exosomes and inhibiting RAP2C expression (Luo et al., 2019).

### Calcification

Calcification and senescence are ubiquitous in older people with AS, high blood pressure, or diabetes. The pathogenesis of coronary calcification is mediated by exosomes derived from VSMCs. In exosomes of a cellular calcification model, target genes of differentially expressed miRNAs were found to be involved in various biological processes, such as development, metabolism, cell composition, tissue biogenesis, as well as signal transduction pathways, such as protein kinase B. The most significant changes in exosomes in the cellular calcification model are the downregulation expression of mmu-let-7e-5p and the upregulation of mmu-miR-324-3p. Calcified cells transfected with miR-324-3p inhibitor regulate protein expression levels, increase IGF1R expression, and decrease PIK3CA and MAP2K1 expression. The expression of these proteins may play a role in reversing calcification (Pan et al., 2020).

Melatonin (MT) affects the cardiovascular system. Exosomes isolated from MT-treated VSMCs or calcified vascular smooth muscle cells (CVSMCs) can be absorbed by VSMCs, and the osteogenic differentiation and senescence of VSMCs or CVSMCs can be inhibited by the paracrine mechanism. It was also confirmed that miR-204/miR-211 in exosomes mediates paracrine effects of exosomes secreted by VSMCs. The exosomes of MT-treated VSMCs alleviate vascular calcification and senescence by utilizing exosomal miR-204/miR-211 (Xu et al., 2020).

## Exosomal Non-Coding RNAs in Coronary Artery Disease Diagnosis

**FIGURE 3:**
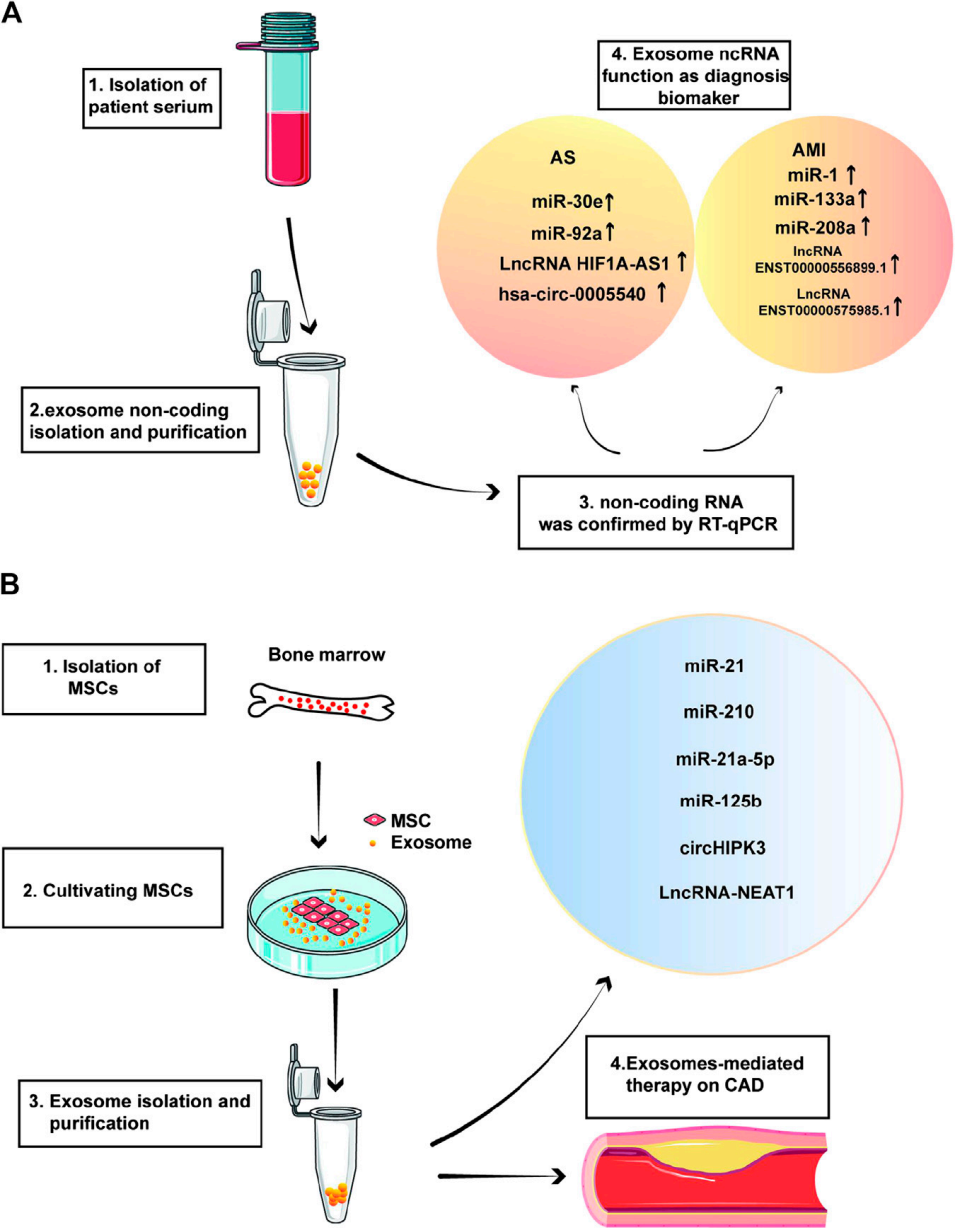
Workflow for diagnostic approaches **(A)** and therapeutic strategy **(B)** of coronary artery disease (CAD) by exosomal non-coding RNAs (ncRNA).

Numerous studies have shown that the exosomes detected in the serum of patients with malignant tumors can be used as a potential biomarker, and the separation and identification of exosomes from body fluids can lead to the development of a valuable diagnostic marker. Studies on the diagnostic value of exosomal ncRNA in CAD are ongoing, and the workflow for diagnostic approaches of CAD is shown in Figure 3A. Most miRNAs isolated from plasma contain exosomes and bind to RNAs, but only a few miRNAs are free (Gallo et al., 2012). Exosomes can be found in a variety of body fluids; therefore, they are promising tools as prognostic and diagnostic tools for pathological diseases (Rezaie et al., 2018).

MiR-30e and miR-92a are overexpressed in plasma exosomes in patients with coronary AS, and the ATP-binding cassette (ABCA1), also known as CERP cholesterol efflux regulatory protein, is the direct target of miR-30e and miR-92a. These results suggest that the level of serum exosomal miR-30e may be a new diagnostic biomarker for coronary AS (Wang Y. et al., 2019; Wang Z. et al., 2019). Wang et al. collected clinical samples and found that the exosome concentration in patients with AS was significantly higher than that in healthy individuals; a similar pattern was also observed in the expression levels of exosome lncRNA HIF1A-AS1. It was speculated that exosome lncRNA HIF1A-AS1 could be used as a potential biomarker of AS (Wang et al., 2017). Another study analyzed circRNA expression in plasma exosomes from patients with CAD and controls using RNA sequencing and found that plasma exosomal hsa_circ_0005540 could be a promising biomarker for the diagnosis of CAD (Wu et al., 2020).

In AMI, the level of circulating cardiac miRNAs is significantly increased, making it a promising diagnostic marker for the early diagnosis of AMI. Studies have found that miR-1, miR-133a, and miR-208a are heart-specific or heart-enriched miRNAs, which can be used as reliable and sensitive markers for the early diagnosis of AMI (Chistiakov et al., 2016). Studies have also revealed that expressions of lncRNAs ENST00000556899.1 and ENST00000575985.1 in circulating exosomes are significantly increased in patients with AMI compared with those in healthy people, which can be used as potential biomarkers to predict the prognosis of patients with AMI (Zheng et al., 2020).

## Therapeutic Application of Exosomal ncRNAs in Coronary Artery Disease

In recent years, stem cell therapy has attracted much attention in many research fields and has been successfully applied in the treatment of many diseases. However, in the cardiovascular field, although the myocardial infarction area can be reduced by stem cell transplantation and myocardial repair and the blood supply can be induced to treat myocardial infarction, the survival rate of stem cell transplantation is very low, and arrhythmia and immune rejection may occur after transplantation; therefore, the therapeutic application of exosomal ncRNAs is not yet feasible (Cambria et al., 2017). To study the cardiac protection and repair effects of exosomes, researchers isolated exosomes from different sources of stem cells. MSCs have been effective in preclinical animal models and clinical trials, but the mechanism of inducing myocardial protection and repair is still not fully understood. Exosomes are considered to be the key mediators of beneficial paracrine effects of MSCs. They have natural stability, low immunogenicity, and toxicity, as well as a high protective effect on their cargo, and they circulate in the whole body to exert their functions. These advantages make exosomes ideal drug carriers (Ha et al., 2016). Exosomes can be loaded with a variety of therapeutic molecules, including miRNAs, siRNAs, mRNAs, and proteins (Wahlgren et al., 2012; Zhang et al., 2017), and the workflow for therapeutic strategy of CAD is shown in Figure 3B.

A study revealed that miR-21 is highly enriched in exosomes derived from BMSCs (Shi et al., 2018). Exosomes carrying miR-21 can be effectively internalized into C-kit^+^ CSCs to protect these cells against apoptosis through reducing PTEN expression and activating PI3K/AKT signaling pathway, and play a protective role in cell death caused by oxidative stress. Therefore, BMSC-derived exosomes can be used as therapeutic vectors to promote c-Kit + CSC in the treatment of ischemic myocardium. Studies have confirmed that BMSC-derived exosomes transfer specific miRNAs to protect cardiac myocytes from apoptotic cell death. Injecting MSC-derived exosomes directly into the heart after coronary artery ligation in rats reduces infarction size and improves cardiac function. *In vitro*, exosomes derived from MSCs improve the survival of cardiomyocytes against hypoxia. Dual-luciferase report analysis showed that miR-210 is the mediator of this therapeutic effect and high expression of miR-210 in MSC exosomes improves the protective effect of cardiomyocytes against stress by targeting AIFM3 (Cheng et al., 2020). Luther et al. confirmed that exosomes in MSCs downregulate the expression of pro-apoptotic gene products PDCD4, PTEN, Peli1, and FasL in the myocardium by increasing the expression of miR-21a-5p in the recipient cardiac myocytes. Using miR-21a mimic transfection treatment and wild-type and miR-21a gene knockout in exosomes derived from MSCs, we confirmed that miR-21a-5p in exosomes is transferred into the myocardium and is the main cardiac protective paracrine factor produced by MSCs through a multi-pathway synergistic action (Luther et al., 2018). Chen et al. demonstrated that exosomes derived from BMSCs highly expressing miR-125b have a protective effect on rat myocardium, reduce cell apoptosis and inflammatory response, and improve cardiac function (Chen et al., 2020). Zhu et al. confirmed that miR-125b-5p in exosomes derived from BMSCs under hypoxic conditions promote ischemic heart repair by improving apoptosis of myocardial cells. Exosomes obtained with low-oxygen-containing covalently and ischemic myocardium-targeted peptides can significantly eliminate apoptosis of ischemic myocardial cells and are expected to become new drug carriers and improve the specificity of drug delivery in ischemic diseases (Zhu et al., 2018). Wang et al. found that the expression of exosomal circHIPK3 derived from hypoxia-induced myocardial cells is upregulated, which promotes angiogenesis and limits the area of myocardial infarction, partly via the miR-29a/VEGFA axis, to maintain myocardial function and integrity of ECs after myocardial infarction and protect the myocardium. Exosomal circHIPK3 in myocardial cells may become a therapeutic target for ischemic injury (Wang et al., 2020). Chen et al. found that lncRNA-NEAT1 in exosomes derived from MIF-pretreated BMSCs plays an anti-apoptotic role by competing with miR-142-3p for FOXO1 activity and may have a protective role in myocardial cell apoptosis (Chen et al., 2020).

## Conclusion and Outlook

We discussed the biogenesis, secretion, isolation, and detection of exosomes, as well as the classification and role of ncRNA, and ncRNA-mediated intercellular communication in CAD. Exosomes are biological information carriers that could change the pathophysiological process of CVD by delivering beneficial or harmful mediators. Among various biomolecules carried by circulating exosomes, ncRNAs are particularly important because of their great regulatory potential. NcRNAs are highly abundant in cells and can affect intracellular signaling pathways, and several ncRNAs could affect specific cellular functions, which highlighting the importance of ncRNAs in the pathophysiology of CVD. Exosomes may serve as clinical delivery carriers of new drugs for CAD. Detailed characterization and functional evaluation of exosomes are necessary to confirm the therapeutic effect of exosomes on CAD. Clarifying exosome cargo and transmitted signals provide valuable information for early diagnosis and treatment. NcRNAs play a broad regulatory role in coronary atherosclerosis and can be transferred to proximal and distal cells through the exosomes to play a biological role. Therefore, specifically targeted anti-AS ncRNAs may be a promising approach for the treatment of CVD.

Much of the current knowledge based on *in vitro* studies and simple evaluation of exosome, such as concentration of samples collected from clinical practice. Related physiological and clinical discoveries were still less than desirable. Thus, exosome-based therapies still require more preclinical studies before translation to human medicine. Recent biodistribution studies have shown that unmodified exosomes rapidly accumulate in the organs involved in the mononuclear macrophage system, such as the liver and spleen, after intravenous injection, and rarely reach the heart after systemic administration (Wiklander et al., 2015). The problems we need to focus on are as follows: 1) New techniques and protocols are needed for the isolation, purification, and detection of exosomes; 2) The poor efficacy of intracoronary administration is a major limitation of the clinical application of exosomes. Therefore, the clinical application of exosomes in the diagnosis and treatment of CAD is promising, but there is still a long way to go.

## Author Contributions

JL and JW researched the article and wrote the manuscript. JW, LL and TL reviewed and edited the manuscript before submission. All authors provided substantial contribution to the discussion of content.

## Funding

This study was supported by Key Scientific and Technological Project of Science and Technology Department of Jilin Province (20170204032YY), Industrial Technology Research and Development Project in Jilin Province (2020C036-3) and the basic and clinical research of coronary heart disease and artificial intelligence diagnosis team in Jilin Province (20200301003RQ).

## Conflicts of Interest

The authors declare that the research was conducted in the absence of any commercial or financial relationships that could be construed as a potential conflict of interest.

## Abbreviations

AMI, Acute myocardial infarction; AS, Atherosclerosis; BMSC, Bone marrow mesenchymal stem cells; CAD, Coronary artery disease; circRNAs, circular ncRNAs; CMVEC, cardiac microvascular endothelial cells; CPC, Cardiac progenitor cells; CSC, Cardiac stem cells; CVD, Cardiovascular disease; CVSMC, Calcified vascular smooth muscle cells; EC, Endothelial cells; ESCRT, Endosomal sorting complexes required for transport; HAOSMC, Human aortic smooth muscle cells; hucMSC, human umbilical cord mesenchymal stem cells; HUVEC, Human umbilical vein endothelial cells; ILVs, intraluminal vesicles; lncRNAs, long ncRNAs; MI, Myocardial infarction; miRNAs, microRNAs; mRNA, Messenger RNA; MSC, Mesenchymal stem cells; MT, Melatonin; ncRNAs, Non-coding RNAs; Vps4, vacuolar protein sorting 4; VSMC, Vascular smooth muscle cell.
